# Erythema Induratum as a rare manifestation of cutaneous tuberculosis in a young woman: a case report

**DOI:** 10.1093/omcr/omaf058

**Published:** 2025-06-27

**Authors:** Harun Iskandar, Yusuf Haz Condeng, Nur Ahmad Tabri, Dewi Setiawati, Diana Muchsin

**Affiliations:** Department of Internal Medicine, Faculty of Medicine, Hasanuddin University, Jalan Perintis Kemerdekaan KM 11, Tamalanrea, Makassar, South Sulawesi, 90245, Indonesia; Department of Internal Medicine, Faculty of Medicine, Hasanuddin University, Jalan Perintis Kemerdekaan KM 11, Tamalanrea, Makassar, South Sulawesi, 90245, Indonesia; Department of Internal Medicine, Faculty of Medicine, Hasanuddin University, Jalan Perintis Kemerdekaan KM 11, Tamalanrea, Makassar, South Sulawesi, 90245, Indonesia; Faculty of Medicine and Health Sciences, Alauddin Makassar State Islamic University, Jalan H. M. Yasin Limpo No. 36, Somba Opu, Gowa Regency, South Sulawesi, 92113, Indonesia; Faculty of Medicine and Health Sciences, Alauddin Makassar State Islamic University, Jalan H. M. Yasin Limpo No. 36, Somba Opu, Gowa Regency, South Sulawesi, 92113, Indonesia

**Keywords:** tuberculosis, cutaneous, erythema Induratum, panniculitis

## Abstract

Cutaneous tuberculosis (TB) is a rare form of extrapulmonary TB, often leading to diagnostic challenges due to its varied clinical presentation. Erythema induratum of Bazin (EIB), a type of lobular panniculitis, is a recognized manifestation of cutaneous TB. This report describes a 19-year-old woman who developed EIB, presenting with recurrent fever, night sweats, weight loss, and hyperpigmented nodular lesions on both lower extremities. Laboratory tests showed an elevated erythrocyte sedimentation rate and a positive tuberculin skin test, while histopathology confirmed lobular panniculitis. Pulmonary TB was excluded based on negative molecular testing and normal chest imaging. The patient was diagnosed with cutaneous TB and treated with WHO-recommended first-line anti-TB therapy, leading to significant clinical improvement. This case underscores the importance of early recognition and timely treatment of cutaneous TB to prevent complications and ensure optimal outcomes.

## Introduction

Tuberculosis (TB) is an infectious disease caused by *Mycobacterium tuberculosis* (MTB), affecting both the lungs (pulmonary TB) and other organs (extrapulmonary TB). Despite global efforts to control TB, an estimated 10.8 million cases were reported in 2023, with the highest prevalence in India (26%), Indonesia (10%), and China (6.8%). The global incidence rate reached 134 cases per 100 000 population [1]. Extrapulmonary TB accounts for approximately 10% of all TB cases, with cutaneous TB being a rare form, representing only 1%–1.5% of extrapulmonary TB cases. Cutaneous TB manifests through hematogenous spread, lymphatic dissemination, or direct inoculation, the latter occurring via skin trauma, wounds, or surgical procedures [2, 3]. Additionally, attenuated MTB from the Bacillus Calmette-Guérin (BCG) vaccine can also contribute to cutaneous TB. Due to its diverse clinical presentations, cutaneous TB often mimics other dermatologic conditions, leading to diagnostic challenges and delays in appropriate treatment initiation [2]. Here, we present the case of a 19-year-old woman diagnosed with erythema induratum, a rare manifestation of cutaneous TB.

## Case report

**Figure 1:**
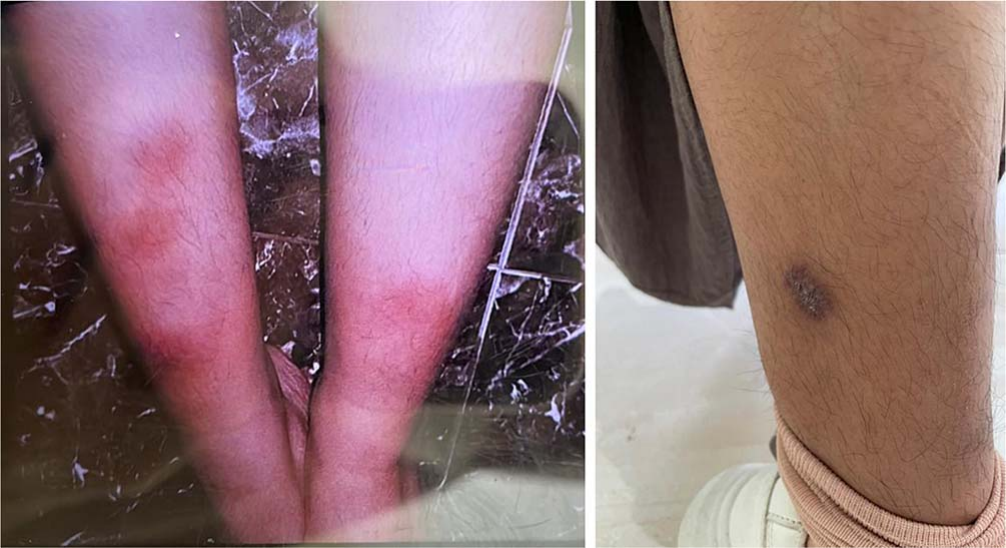
Hyperpigmented lesions on the bilateral lower extremities.

A 19-year-old female presented with a 3-month history of recurrent fever, predominantly occurring in the evenings and at night. She reported frequent night sweats and unintentional weight loss of 4 kg over the same period. Additionally, she developed red rashes on both legs, which gradually enlarged, darkened from red to black, and were accompanied by wounds. The lesions were itchy, painful, and non-healing. The patient denied respiratory symptoms such as cough, hemoptysis, or shortness of breath. While she reported no known history of tuberculosis contact initially, further inquiry revealed contact with a confirmed TB patient. She had no prior history of antituberculosis drugs (ATD) use, BCG vaccination, allergies, or previous episodes of skin rashes. On examination, the patient had a mild pyrexia of 37.9°C. Clinical examination revealed no lymphadenopathy, and the chest examination was unremarkable. The skin lesions appeared as hyperpigmented macules on both lower extremities (Fig. 1).

**Figure 2:**
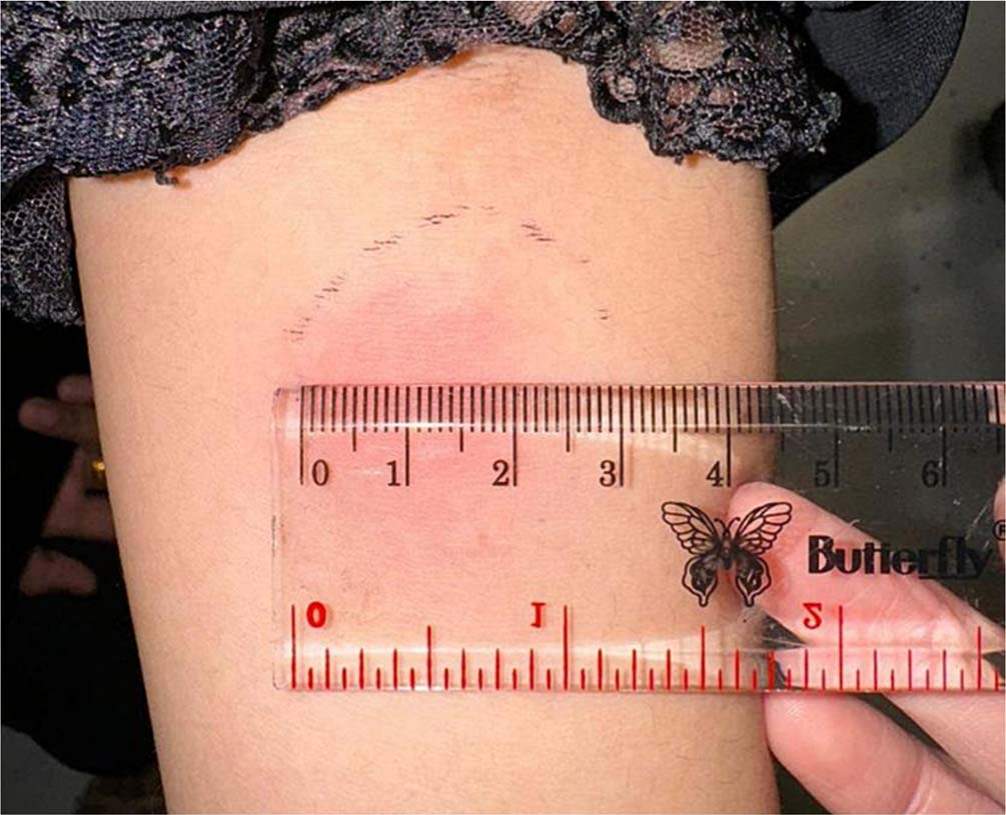
Tuberculin skin test (TST) result showing a 23 mm induration (positive).

**Figure 3:**
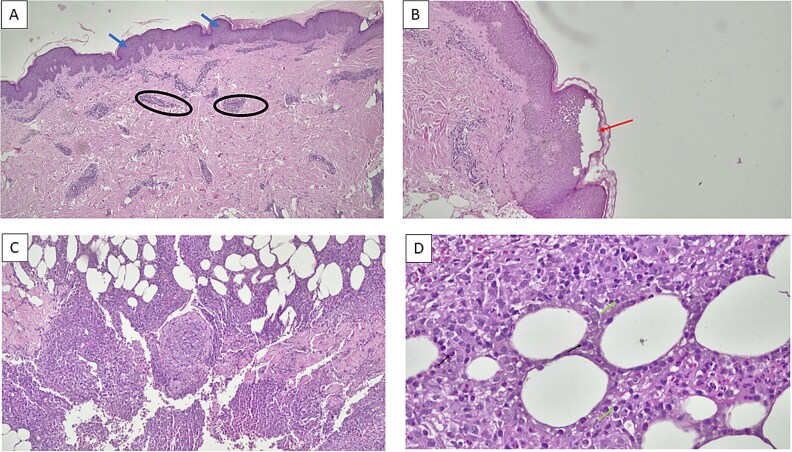
Histopathological results. (A) Epidermal acanthosis in the dermis, with perivascular inflammation observed in the superficial, mid, and deep dermis. Inflammatory cells include lymphocytes, plasma cells, neutrophils, and eosinophils (objective 4x); (B) The section shows skin with a spongiotic reaction at 4x magnification; (C) Inflammation extends to the subcutaneous fat, resulting in lobular panniculitis (10x magnification); (D) Inflammatory cells consist of lymphocytes, plasma cells, neutrophils, and eosinophils. The inflammation extends to the subcutaneous fat, resulting in lobular panniculitis (objective 40x).

Routine blood tests showed an elevated erythrocyte sedimentation rate of 62 mm/hour. The induced sputum rapid molecular test did not detect MTB, and the thoracic X-ray showed no signs of pulmonary tuberculosis. Further diagnostic testing included a tuberculin skin test (TST), where 0.1 ml containing 5 TU PPD was injected into the arm, and the results were interpreted after 48 h. The TST result showed a 23 mm induration, indicating a positive result (Fig. 2). A skin biopsy was performed in this patient, with the results (Fig. 3) showing lobular panniculitis, consistent with erythema induratum.

**Figure 4:**
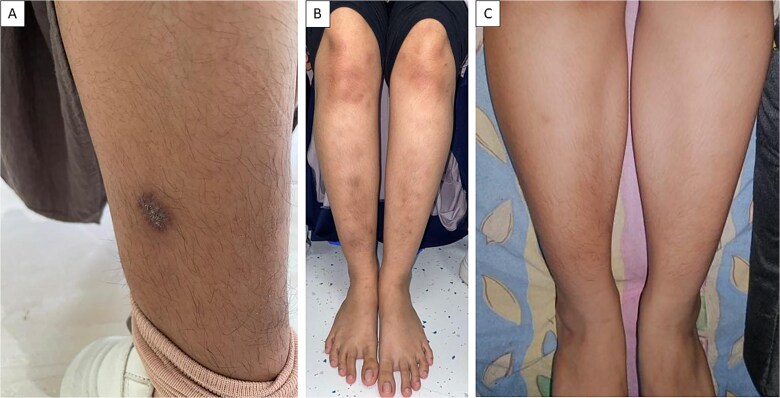
Skin lesion progression. (A) Before the initiation of anti-tuberculosis drugs (ATD); (B) Two months after starting ATD; (C) Six months after OAT, showing complete healing of the skin lesions.

Routine follow-up was conducted at the clinic before starting anti-tuberculosis therapy, after two months, and at the six-month mark. In this patient, anti-tuberculosis drugs (ATD) were administered as 4FDC 1x4 tablets, with each tablet containing 150 mg Rifampicin, 75 mg Isoniazid (INH), 400 mg Pyrazinamide, and 275 mg Ethambutol. After initiating therapy, the patient no longer experienced fever or night sweats, and her weight increased by 2 kg. The skin lesions gradually improved, and the wounds healed (Fig. 4).

## Discussion

Extrapulmonary tuberculosis accounts for approximately 10% of all tuberculosis cases, with cutaneous tuberculosis comprising about 1% to 1.5% of extrapulmonary TB cases [3]. Cutaneous tuberculosis is a skin infection caused by MTB. The cutaneous invasion of MTB can occur either endogenously or exogenously. Endogenous invasion typically results from the spread of pulmonary tuberculosis through hematogenous or lymphatic dissemination, whereas exogenous invasion occurs through direct inoculation of the bacteria [3, 4]. In this case, the cutaneous TB developed due to close contact with the patient’s father, who was positive for TB. The patient presented no respiratory symptoms, such as cough, and the chest X-ray showed no signs of pulmonary tuberculosis, indicating that the pathogenesis was likely due to direct inoculation of MTB through a wound on the foot.

In this patient, clinical manifestations included systemic symptoms such as intermittent fever and night sweats. The fever was low-grade, ranging from 37.3 to 38°C. Tuberculosis induces fever as the MTB bacteria trigger an immune response, leading to the release of cytokines and vascular endothelial growth factor (VEGF), which promote angiogenesis and increase vascularization in the affected lesions. Night sweats are a common symptom of TB, resulting from the immune system’s response to MTB. This response involves the release of cytokines, such as interleukin-6 (IL-6), tumor necrosis factor-alpha (TNF-α), and interferon gamma (IFN-γ), which disrupt body temperature regulation, particularly during the night, causing excessive sweating [5, 6].

A significant laboratory finding in this case was the elevated erythrocyte sedimentation rate (ESR) of 62 mm/hour. A high ESR is commonly observed in patients with tuberculosis, indicating the presence of systemic inflammation. Tuberculosis is a chronic infection that triggers the release of inflammatory cytokines into the bloodstream. Approximately 46% of patients with active TB exhibit an ESR > 60 mm/hour, while 40% have levels between 40–60 mm/hour [7]. This patient presented with non-healing skin lesions, including hyperpigmented macules on the bilateral lower extremities. The clinical manifestations of cutaneous TB are diverse and complex, often resembling various other skin conditions, which complicates diagnosis. Cutaneous TB may present as spots, plaques, macules, papules (including papulonecrotic, papuloerythematous, and papulocrustous forms), nodules, erythema induratum, erosion, and scrofuloderma, all of which can mimic a range of other dermatologic diseases [8, 9].

Early cutaneous TB lesions may exhibit surface ulceration, epidermal hyperplasia, and dense dermal infiltration by neutrophils, lymphocytes, and plasma cells. Over time, chronic inflammatory cells and granulomatous inflammation replace the neutrophils, with varying degrees of caseous necrosis. In advanced cases of cutaneous TB, acid-fast bacilli are rarely observed following the development of granulomas. In this case, histopathological examination of the skin biopsy revealed mild spongiotic changes and epidermal acanthosis. Perivascular inflammation was noted in the superficial, mid, and deep dermis, with inflammatory cells including lymphocytes, plasma cells, neutrophils, and eosinophils. The inflammation extended into the subcutaneous fat, resulting in lobular panniculitis consistent with erythema induratum, a characteristic finding in the early stages of cutaneous tuberculosis [10, 11].

Erythema induratum of Bazin (EIB) and nodular vasculitis (NV) are types of lobular panniculitis, with clinical manifestations primarily involving the lower extremities. These conditions typically affect young to middle-aged women, although they are rarely seen in children. In some cases, the identification of *M. tuberculosis* DNA through PCR testing on skin lesions helps confirm the tuberculosis etiology. Additionally, the tuberculin skin test is usually positive in such cases [12, 13].

The diagnosis of cutaneous TB in this patient was based on symptoms of intermittent fever, night sweats, and weight loss over three months. Supporting examinations revealed an elevated ESR and a positive tuberculin skin test with a diameter of 23 mm. The diagnosis was further confirmed through tissue biopsy and Mantoux test, with biopsy results showing inflammatory cells including lymphocytes, plasma cells, neutrophils, and eosinophils. The inflammation extended to the subcutaneous fat, resulting in lobular panniculitis, which was consistent with erythema induratum [14, 15].

In this patient, a 4FDC Anti-Tuberculosis Drug therapy was initiated in April 2024, consisting of 3 tablets daily for the first 2 months of the intensive phase, followed by 4 months of the continuation phase. After one month of treatment, significant improvements were observed in the lesion area, and the patient experienced no side effects from the medication. The prognosis for cutaneous TB is generally favorable in patients with a healthy immune system. However, in immunocompromised individuals, even with aggressive treatment, the disease may persist, and the organism may develop resistance to multiple drugs.

## Summary

This case report describes a 19-year-old woman with cutaneous tuberculosis presenting as erythema induratum, a rare form of lobular panniculitis. She experienced fever, night sweats, weight loss, and non-healing skin lesions, but no respiratory symptoms. Diagnosis was confirmed through a positive tuberculin skin test and biopsy showing lobular panniculitis. The condition was treated with a four-drug anti-tuberculosis regimen, resulting in significant improvement. This case highlights the importance of early recognition and treatment of rare cutaneous TB manifestations like erythema induratum.
